# The Bio-Synthesis of Three Metal Oxide Nanoparticles (ZnO, MnO_2_, and MgO) and Their Antibacterial Activity Against the Bacterial Leaf Blight Pathogen

**DOI:** 10.3389/fmicb.2020.588326

**Published:** 2020-12-04

**Authors:** Solabomi Olaitan Ogunyemi, Muchen Zhang, Yasmine Abdallah, Temoor Ahmed, Wen Qiu, Md. Arshad Ali, Chengqi Yan, Yong Yang, Jianping Chen, Bin Li

**Affiliations:** ^1^State Key Laboratory of Rice Biology, Ministry of Agriculture Key Lab of Molecular Biology of Crop Pathogens and Insects, Institute of Biotechnology, Zhejiang University, Hangzhou, China; ^2^Department of Crop Protection, Federal University of Agriculture Abeokuta, Abeokuta, Nigeria; ^3^Department of Plant Pathology, Faculty of Agriculture, Minia University, Minya, Egypt; ^4^Institute of Plant Virology, Ningbo University, Ningbo, China; ^5^State Key Laboratory for Managing Biotic and Chemical Threats to the Quality and Safety of Agro-products, Institute of Virology and Biotechnology, Zhejiang Academy of Agricultural Sciences, Hangzhou, China

**Keywords:** antibacterial, biosynthesis, nanoparticles, *Paenibacillus polymyxa*, *Xanthomonas oryzae* pv. *oryzae*

## Abstract

*Xanthomonas oryzae* pv. *oryzae* (*Xoo*) is the most infectious pathogen of rice, which causes bacterial leaf blight (BLB) disease. However, the accumulation of chemical or antibiotic resistance of *Xoo* necessitate the development of its alternative control. In this study, we biologically synthesize three metal oxide nanoparticles (ZnO, MnO_2_, and MgO) using rhizophytic bacteria *Paenibacillus polymyxa* strain Sx3 as reducing agent. The biosynthesis of nanoparticles was confirmed and characterized by using UV-vis spectroscopy, XRD, FTIR, EDS, SEM, and TEM analysis. The UV Vis reflectance of the nanoparticle had peaks at 385, 230, and 230 nm with an average crystallite particle size 62.8, 18.8, and 10.9 nm for ZnO, MnO_2_, and MgO, respectively. Biogenic ZnO, MnO_2_, and MgO nanoparticles showed substantial significant inhibition effects against *Xoo* strain GZ 0006 at a concentration of 16.0 μg/ml, for which the antagonized area was 17, 13, and 13 mm and the biofilm formation was decreased by 74.5, 74.4, and 80.2%, respectively. Moreover, the underlining mechanism of nanoparticles was inferred to be in relation to the reactive oxygen species based on their antibacterial efficiency and the deformity in the cell wall phenomenon. Overall, an attractive and eco-friendly biogenic ZnO, MnO_2_, and MgO nanoparticles were successfully produced. Altogether, the results suggest that the nanoparticles had an excellent antibacterial efficacy against BLB disease in rice plants, together with the increase in growth parameter and rice biomass. In conclusion, the synthesized nanoparticles could serve as an alternative safe measure in combatting the antibiotic-resistant of Xoo.

## Introduction

*Xanthomonas oryzae* pv. *oryzae* (*Xoo*) is affiliated to the gamma subdivision of Gram-negative proteobacterial with a single polar flagellum (Yang et al., 2007; Lee et al., 2008). It is rod-shaped having light yellow, circular and smooth colonies when grown on nutrient agar media (Jonit et al., 2016). For the biochemical test, most strains of *Xoo* showed positive reaction against the catalase test and adverse reaction for the oxidase test, while some strains varied in their reaction to starch hydrolysis (Swings et al., 1990; Samanta et al., 2014). Bacterial leaf blight (BLB) caused by *Xoo* is among the most destructive rice diseases occurring in the rice-growing areas of the world with a huge crop loss of approximately 50% of the economic plant part (Sharma P. et al., 2017). Several management strategies have been used to combat this plant pathogen, but each has been met with certain drawbacks. Nanoparticles (NPs) have been successfully applied in the agriculture, health and food sector, providing eco-friendly alternative strategies for managing BLB (Peters et al., 2014).

The synthesis of NPs have been carried out by physical, chemical, or biological methods. The drawbacks of the physical and chemical methods are the high intake of energy needed to meet the requirement of the high temperature and pressure for NPs synthesis and the toxic byproducts released to the environment is unavoidable (Zhou et al., 1999; Chen et al., 2003). Therefore, researchers are faced with the need of the hour to produce high yielding, low cost, non-toxic and eco-friendly metallic nanoparticles (Thakkar et al., 2010). In order to synthesize eco-friendly and biocompatible nanoparticles, green synthesized materials have been widely adopted (Borase et al., 2014). Among the reported metal oxides, zinc oxide (ZnO) and magnesium oxide (MgO) nanoparticles have accrued much popularity because of their stability during harsh process conditions and their safety properties to human health (Stoimenov et al., 2002; Jones et al., 2008; Sundrarajan et al., 2012). Diverse materials have been used in the biosynthesis of NPs including bacteria, plants, microalgae, lichen, actinomycetes, yeasts, and fungi (Ahmad et al., 2003; Mourato et al., 2011; Salunkhe et al., 2011; Castro et al., 2013; Mie et al., 2014; Pandian et al., 2015; Fouad et al., 2017; Ogunyemi et al., 2019a, b).

Magnesium oxide nanoparticles (MgONPs) have been accounted to serve as a catalyst due to its affordability, antibacterial agent, and biodiesel synthesis (Montero et al., 2010; Mirzaei and Davoodnia, 2012; Tang and Lv, 2014). Manganese dioxide nanoparticles (MnO_2_NPs) have been used in medicine, catalysis, ion-exchange, adsorption, sensor, and energy sectors (Moon et al., 2015), while zinc oxide nanoparticles (ZnONPs) are used as pathogenic microbes inhibitor and its antibacterial activity against Gram-negative and Gram-positive bacteria are well documented (Lakshmi et al., 2012; Divyapriya et al., 2014).

Earlier studies have reported the synthesis of metal oxide nanoparticles using the extracellular components of *Bacillus* sp., *Escherichia coli, Ureibacillus thermosphaericus, Corynebacterium glutamicum*, and *Lactobacillus* sp. (Bao et al., 2010; Jha and Prasad, 2010; Sneha et al., 2010; Juibari et al., 2015; Fouad et al., 2017). The mechanism of silver nanoparticles formation by bacterial cell culture was proposed to be the involvement of enzyme nitrate reductase (Kalimuthu et al., 2008), while Fouad et al. (2017) reported that the protein in the cell-free supernatant of *Bacillus* sp. was responsible for the reduction of AgNPs. Extracts of living organisms serve as reducing and capping agents (Ahmad et al., 2003; Castro et al., 2013; Fouad et al., 2017; Rangarajan et al., 2018), and they are the most attractive and simple sources for the production of NPs (Mourato et al., 2011; Borase et al., 2014). The effectiveness of lipopeptides or secondary metabolites of microorganisms as capping and stabilizing agents for the biosynthesis of silver nanoparticles have been reported in *Bacillus* (Rangarajan et al., 2018) and *Pseudomonas aeruginosa* (Kumari et al., 2017). The Gram-positive bacteria *Paenibacillus polymyxa* was reported to produce secondary metabolites such as fusaricidins and polymyxin, a family of lipopeptides and amylase (Abdallah et al., 2019a), the possibility to be applied for nanoparticles biosynthesis still awaits investigation.

The application of green synthesis MgO nanoparticles is still unexplored, while manganese dioxide (MnO_2_) and ZnO nanoparticles green synthesis are also not fully explored. These nanoparticles are non-toxic and are promising antimicrobial agents in the agricultural field. Therefore, this study was carried to synthesize MgO, MnO_2_, and ZnO nanoparticles using rhizophytic bacteria *P. polymyxa* strain Sx3, which produces secondary metabolites and enzymes reduction and capping of nanoparticles. The resultant nanoparticles were further tested for their antibacterial efficiency against *X. oryzae* pv. *oryzae* strain GZ 0006, the causal pathogen of bacterial leaf blight *in vitro* and *in vivo*.

## Materials and Methods

### Collection of Bacterial Strain and Culture Conditions

**FIGURE 1:**
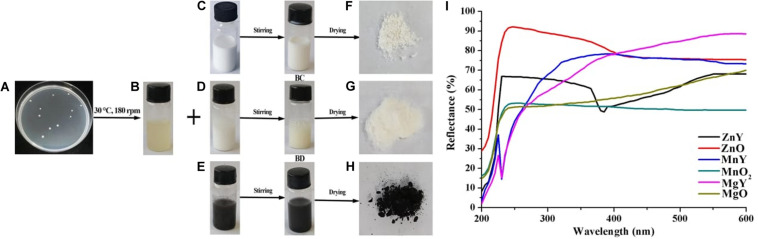
Schematic presentations of synthesis of ZnO, MnO_2_, and MgO nanoparticles. **(A)** Colony of *Paenibacillus polymyxa* strain Sx3 **(B)** Supernatant of *P. polymyxa* strain Sx3 **(C)** ZnO solution **(D)** MgO solution **(E)** MnO_2_. solution **(B,C)** ZnO nanoparticles **(B,D)** MgO nanoparticles **(B,E)** MnO_2_ nanoparticles **(F)** Freeze-dried ZnO nanoparticles **(G)** Freeze-dried MgO nanoparticles **(H)** Freeze-dried MnO_2_ nanoparticles and **(I)** UV-Vis reflectance spectra of bulk metal oxides of ZnO, MgO, and MnO_2_ and synthesized ZNY–ZnO nanoparticles, MNY–MnO_2_ nanoparticles, and MGY–MgO nanoparticles.

The fresh culture of *P. polymyxa* strain Sx3 was collected from the laboratory collection of State key laboratory of rice Biology, Institute of Biotechnology, Zhejiang University, China. A colony of the bacteria (Figure 1A) was cultured overnight in 5 ml nutrient broth, 3 ml of the grown bacteria was transferred to 300 ml of nutrient broth shaken at 180 rpm/min at 30°C for 24 h. The cultured bacteria was centrifuged at 5,000 g for 5 min, and the supernatant that don’t have antibacterial activity again Xoo strain GZ 0006 was collected and used to synthesize the nanoparticles.

### Extracellular Biosynthesis of Metal Oxide NPs

Zinc oxide, magnesium oxide, and manganese dioxide were purchased from Sigma-Aldrich (>99% purity). The metal oxides NPs were synthesized with slight modifications according to Rajabairavi et al. (2017). Briefly, 100 ml of 5 × 10^8^ CFU/ml of the cell-free supernatant of *P. polymyxa* strain Sx3 (Figure 1B) gotten through double filter by using 0.22 μm millipore filter and 100 ml each of ZnO, MgO, and MnO_2_ (1 mM) were mixed in their respective flasks. The flask containing the mixture was placed on the magnetic stirrer at room temperature for 24 h. The synthesized ZnO, MnO_2_, and MgO nanoparticles were purified by centrifuging at 10,000 g for 20 min, followed by repetitive centrifugation after resuspending the pellets in double-distilled water. The collected pellets were freeze-dried using Alpha 1–2 LDplus (Model number: 101521, Fisher scientific, United States) and used for further characterization.

### Characterization of Metal Oxide NPs

The bulk metal oxides and the purified nanoparticles of ZnO, MgO, and MnO_2_ were used for characterization. FT-IR spectra (Fourier Transform Infrared Spectrometer) of the functional groups were recorded on Vector 22, Bruker, Bremen, Germany spectrophotometer in the range of 4,000–400 cm^–1^ at room temperature with the resolution of 4 cm^–1^. UV–Vis reflectance spectroscopy of the nanoparticles was determined using UV–Vis spectrophotometry (Shimadzu spectrometer, Kyoto, Japan) in the wavelength range of 200–600 nm. The size and external morphology of the metal oxide nanoparticles and the bulk metal oxide were characterized by SEM using TM-1000, Hitachi, Tokyo, Japan which was equipped with an energy dispersive spectrum (EDS). Transmission Electron Microscopy (TEM) using JEM-1230, JEOL, Akishima, Japan was used to study the structure of the nanoparticles. Crystal phase identification of the metal oxides was characterized by XRD, XPert PRO diffractometer (Holland) with a current of 30 mA using CuKα radiation with 2O ranging from 20 to 80°. The nanoparticle size was estimated by Scherrer’s formula (Cullity, 1978).

### Antibacterial Activity of Metal Oxide NPs

The antibacterial performance of the bulk metal oxides and ZnO, MgO, and MnO_2_ nanoparticles were examined against *Xoo* strain GZ 0006 (Genbank accession: MH 158522) by Minimum inhibitory concentration (MIC) and diffusion methods. The MIC was performed with slight modifications, according to Wiegand et al. (2008). Briefly, 100 μl of twofold serial dilution of an overnight bacterial culture of 10^8^ CFU/ml were mixed with ZnO, MgO, and MnO_2_ at concentrations 4.0, 8.0, and 16.0 μg/ml in a 96-well microtiter plates (Corning-Costar Corp., Corning, NY, United States) and placed in a 30°C incubator for 48 h. Each concentration had three replicates. Wells, which contained only *Xoo* strain GZ 0006 without nanoparticles and bulk metal oxide addition served as the control. The MIC of the of the nanoparticles was read by a Scanning Microplate Spectrophotometer (Thermo Fisher Scientific Inc., Waltham, MA, United States) at OD_6__00_. The diffusion test of the bulk metal oxides and nanoparticles was tested against *Xoo* strain GZ 0006 on a plate assay (Ogunyemi et al., 2019b). Ten microliter concentrations 4.0, 8.0, and 16.0 μg/ml of ZnO, MnO_2_, and MgO nanoparticles and bulk metal oxides were dropped in their respective spots and incubated at 30°C for 18 h. The antibacterial effect was estimated by taking a record of the growth inhibition zones in millimeters. The experiment was conducted in triplicate.

### Swarming Motility Assay

Swarming motility assay was evaluated according to Rashid and Kornberg (2000). Different concentrations of ZnO, MgO, and MnO_2_ nanoparticles and bulk metal oxides (4.0, 8.0, and 16.0 μg/ml) was added to approximately 5 ml of 0.3% semi-solid agar, respectively, then the mixture was turned into individual petri-dishes and cooled. 10 μl of the bacterial culture (10^8^ CFU/ml) of strain GZ 0006 was spotted at the center of the Petri-dishes and incubated at 30°C for 24 h and pure semi-solid agar used as the control. Swarming motility was evaluated by taking a record of the diameter of the bacterial colony.

### Biofilm Inhibition Assay

Effect of the bulk metal oxides and nanoparticles on biofilm formation of *Xoo* strain GZ 0006 was determined using a 96-well plate according to Hassan et al. (2011) with little modifications. In brief, 100 μl of overnight bacterial culture (10^8^ CFU/ml) were dropped into wells of 96-well plate with different concentrations of the bulk metal oxides and nanoparticles (4.0, 8.0, and 16.0 μg/ml) and wells with pure bacterial culture used as the control. The cultures were discarded, washed thrice with sterile water and air-dried and then incubated the plates at 30°C for 48 h. Aqueous crystal violet (>97% purity, Sigma-Aldrich, United States) (100 μl) was added to each well to stain the attached bacterial cells for 30 min. The dye was discarded, and the wells were gently washed off the dye. A hundred microtiter of 33% acetic acid (Sigma-Aldrich, United States) was added into each well, and the intensity was read at OD570 nm.

### Adsorption on the Cell Surface

The preparation of bacterial cells to view the damage caused by the addition of nanoparticles was determined according to Helander et al. (2001). Approximately 10^8^ CFU/ml of *Xoo* strain GZ 0006 was centrifuged at 11,000 g for 10 min, and the pellet was mixed with 8.0 μg/ml of ZnO, MnO_2_, and MgO nanoparticles, respectively. The suspension was incubated in a 30°C for 20 min followed by centrifugation at 11,000 g for 10 min. The samples were then prepared following standard procedures for fixation and embedding. The stained samples were viewed by a JEM-1230 transmission electron microscope (JEOL, Tokyo, Japan) at an operating voltage of 75 kV.

### ROS Production

Relative reactive oxygen species (ROS) produced in *Xoo* strain GZ 0006 was determined using dichlorofluorescein diacetate (DCFH-DA) (≥97% purity, Sigma-Aldrich, United States), a detection reagent for detecting the intracellular ROS as described by Cai et al. (2018) with slight modifications. About 1 ml of overnight-cultured *Xoo* was centrifuged at 5,000 g for 4 min, and the bacterial cells were afterward grouped into three sets. The first set was treated with 16.0 μg/ml of the tested nanoparticles. The second set was treated with nanoparticles in the presence of specific ROS scavenger, rotenone (≥95% purity, Sigma-Aldrich, United States), which served as a negative control (Li et al., 2015), and another control was maintained by treating the bacterial cells with double distilled water. After an incubation time of 4 h, the suspension was washed thrice with phosphate-buffered saline solution and incubated in the dark at 30°C for 30 min after the addition of 10 μM DCFH-DA. A wash step was repeated post-incubation, and the fluorescence was determined using confocal laser microscopy (Zeiss LSM 780, United States). The experiment was performed in the dark because of the specific ROS scavenger, rotenone, which decomposes and oxidizes when exposed to light.

### *In vivo* Inhibitory Effect of ZnO, MgO, and MnO_2_ Nanoparticles on *Xoo* Strain GZ 0006

The *in vivo* inhibitory effect of ZnO, MgO, and MnO_2_ nanoparticles at 16.0 μg/ml on Xoo strain GZ 0006 was evaluated on rice plants. The concentration 16.0 μg/ml was chosen for this experiment because of its high *in vitro* inhibitory effect. After germination, seeds of rice (cultivar II You023, *Oryza sativa* L.) were sown in plastic pots arranged in a completely randomized block design (CRD) in a growth chamber (28 ± 2°C, 80% humidity with 16:8 h light-dark photoperiod). At the third to fourth leaf-stage, respective leaves were inoculated with *Xoo* strain GZ 0006 (10^8^ CFU/ml) or double distilled water by leaf clipping (Kauffman et al., 1973; Ogunyemi et al., 2019c). The plants were treated by foliar spray according to the method of Yasmin et al. (2017) with 16.0 μg/ml of ZnO, MgO, and MnO_2_ nanoparticles, respectively, 24 h post-inoculation. Each treatment had three replicates. The percentage disease leaf area (%DLA) was assessed 14 days post-inoculation by measuring the lesion leaf length relative to the total length of the leaf (Yasmin et al., 2017).

### Statistical Analysis

All results were expressed as mean ± SD (Standard Deviation) of experiments performed in triplicate. The ANOVA test was done using SAS software (SAS, Institute, Cary, United States) and the means were compared by the least significant difference (LSD) method at *P* < 0.05.

## Results

### Biosynthesis of Metal Oxide NPs

Solutions of ZnO, MgO, and MnO_2_ (Figures 1C–E) were prepared to a final concentration of 1 mM, white color was obtained for both ZnO and MgO (Figures 1BC,BD), and black color was gained for MnO_2_ (Figure 1BE). *P. polymyxa* strain Sx3 culture filtrate was added to the prepared ZnO, MgO, and MnO_2_ solutions and applied to magnetic stirring for 24 h, the treatment without culture filtrate was used as the control. As a result, a final product of pale white nanoparticle was present for ZnO and MgO after freeze-drying, and light black color was showed for MnO_2_ nanoparticle after the synthesis process (Figures 1F–H).

### Characterization of Metal Oxide NPs

**FIGURE 2:**
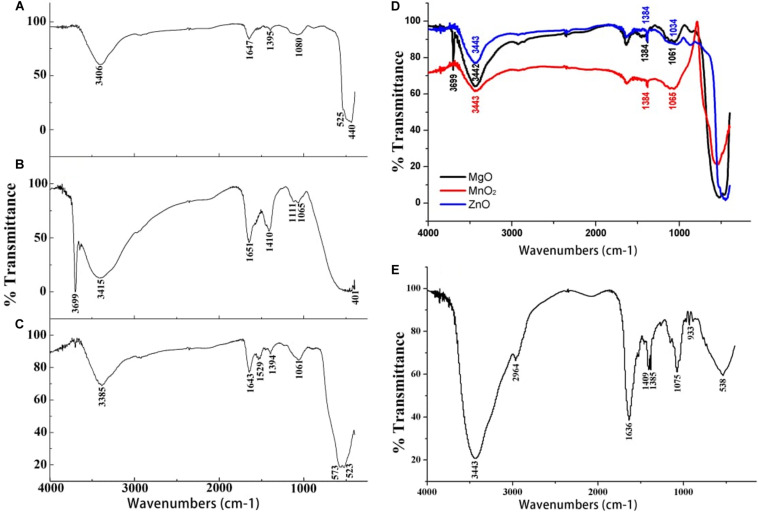
Fourier transform infrared spectra of **(A)** ZnO nanoparticles **(B)** MgO nanoparticles **(C)** MnO_2_ nanoparticles **(D)** Bulk metal oxide of ZnO, MgO, and MnO_2_ and **(E)** Culture filtrate of *P. polymyxa* strain Sx3.

The successful nanoparticles formation were confirmed by the peaks observed in the UV-Vis spectroscopy of the respective nanoparticles which were absent in the bulk counterpart (Figure 1I). The optical reflectance spectra of ZnO, MgO, and MnO_2_ synthesized by *P. polymyxa* strain Sx3 were recorded in a range of 200–600 nm. Optimum peaks at 385, 230 and 230 nm were recorded for ZnO, MnO_2_, and MgO nanoparticles, respectively (Figure 1I). In the UV-Vis reflectance spectra for the bulk solution of ZnO, MgO, and MnO_2_, no maximum peaks were observed (Figure 1I). The functional groups contained in the bulk metal oxide of ZnO, MgO, and MnO_2_, synthesized ZnO, MnO_2_ and MgO materials, and culture filtrate of *P. polymyxa* strain Sx3 are shown in Figure 2. The absorption peaks for ZnO nanoparticles were 3,406, 1,647, 1,395, 1,080, 525, and 440 cm^–1^ (Figure 2A). The absorption peaks at 3,699, 3,415, 1,651, 1,410, 1,111, 1,065, and 401 cm^–1^ were for MgO nanoparticles (Figure 2B), while the absorption peaks for MnO_2_ nanoparticles were measured at 3,385, 1,643, 1,529, 1,394, 1,061, 573, and 523 cm^–1^ (Figure 2C). The absorption peaks common to the bulk metal oxides is 1,334 cm^–1^ which responds to the different modes of CO_2_^–2^ (Figure 2D).

The absorption peaks of FTIR spectra of the nanoparticles at 1,647, 1,651, and 1,643 cm^–1^ is the stretching vibration of C = O due to the amide 1 group, stretching vibration of C = C, and the bending vibration of the N–H bond of primary amine. The peak at 1,529 cm^–1^ is the N–H bending vibration and the stretching vibration of C = C bond. The peaks at 1,061 and 1,065 cm^–1^ are the stretching vibration of the C–N and C–O bond, while the peak at 1,080 cm^–1^ is stretching vibration of C–N bond (Figures 2A–C). The bands at 525 and 440 cm^–1^ are the characteristic bands of the zinc oxide. The peak at 401 cm^–1^ indicates the presence of MgO, while the peaks at 573 and 523 cm^–1^ are the characteristic bands of the manganese dioxide nanoparticles (Figures 2A–C). The absorption peaks at 1,636, 1,409, 1,385, and 1,075 cm^–1^ of the culture filtrate of *P. polymyxa* strain Sx3 imply the stretching of C = C, N–H bending, C–H bending, and stretching of N–O, C–O, and C–O–C, respectively (Figure 2E).

**FIGURE 3:**
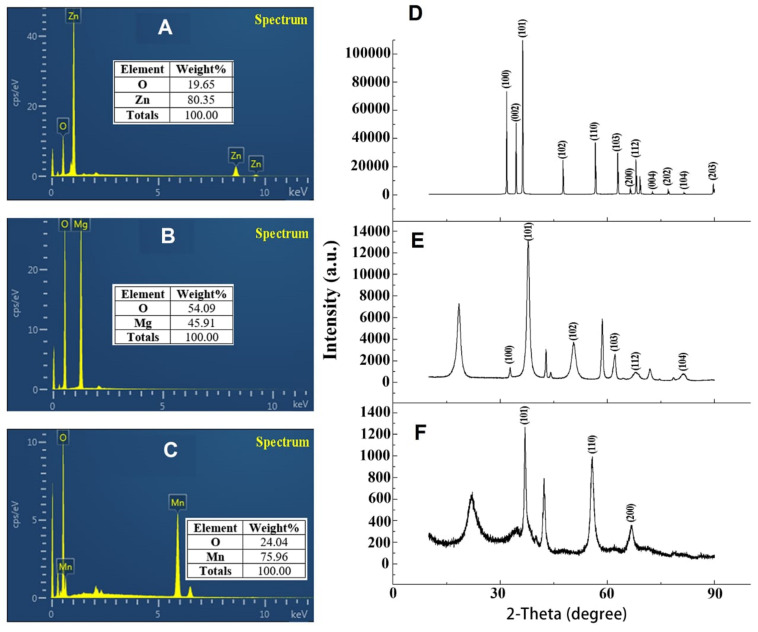
Energy dispersive spectrum profiles of **(A)** ZnO nanoparticles **(B)** MgO nanoparticles **(C)** MnO_2_ nanoparticles and X-ray Diffractometer spectra of **(D)** ZnO nanoparticles **(E)** MgO nanoparticles **(F)** MnO_2_ nanoparticles.

The percentage elemental composition of ZnO, MnO_2_, and MgO nanoparticles synthesized by *P. polymyxa* strain Sx3 were confirmed by energy dispersive spectra (EDS) attached to the scanning electron microscopy (SEM). The nanoparticles synthesized by *P. polymyxa* strain Sx3 were highly intense with maximum intensity at 1.0, 1.5 and 6.0 keV for Zn, Mg, and Mn, respectively, which were highly purified according to the SEM-EDS containing only the respective elements without any other contaminated element (Figures 3A–C). The x-ray diffractive (XRD) patterns of ZnO, MnO_2_, and MgO nanoparticles synthesized by *P. polymyxa* strain Sx3 are shown in Figures 3D–F. The diffraction patterns for ZnO nanoparticles had peaks at 31.83, 34.46, 36.31, 47.60, 56.64, 62.91, 66.43, 67.99, 72.61, 77.01, 81.43, and 88.65° which corresponds to crystal planes of (100), (002), (101), (102), (110), (103), (200), (112), (004), (202), (104), and (203) (Figure 3D). The MgO nanoparticles had its diffraction peaks at 32.81, 37.84, 50.56, 62.18, 68.81, and 87.71° of 2O, which corresponds to (100), (101), (102), (103), (112), and (104) crystal planes (Figure 3E). On the other hand, the XRD patterns for MnO_2_ nanoparticles shows only three diffraction bands at 36.94, 55.80, and 66.68° of 2O which correspond to (101), (110), and (200) crystal planes (Figure 3F). The estimated crystallite size of the synthesized ZnO, MnO_2_, and MgO nanoparticles was determined as an average using Debye Scherrer’s equation (Cullity, 1978) were found to be 62.8, 18.8, and 10.9 nm, respectively, for ZnO, MnO_2_, and MgO nanoparticles synthesized by *P. polymyxa* strain Sx3 (Figures 3D–F).

**FIGURE 4:**
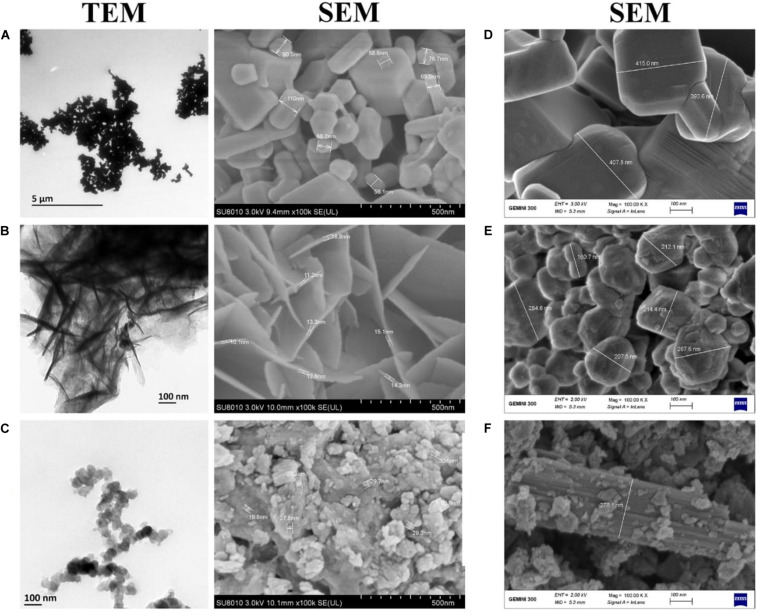
Transmission and Scanning electron micrographs of **(A)** ZnO nanoparticles **(B)** MgO nanoparticles **(C)** MnO_2_ nanoparticles and Scanning electron micrographs of Bulk metal oxides of **(D)** ZnO **(E)** MgO **(F)** MnO_2_. TEM Magnification 5,000X.

The transmission electron microscopy (TEM) images of ZnO, MnO_2_, and MgO nanoparticles are shown in Figures 4A–C. The general structural observations of the ZnO nanoparticles were cubic, MgO nanoparticles had a sheet-like structure, and MnO_2_ had a spherical shape (Figures 4A–C). To validate the structural characteristics of the nanoparticles, SEM was applied, and the sample sizes were estimated. It was confirmed that ZnO nanoparticles were cubic structured with a size range of 56.1–110.0 nm (Figure 4A), the MgO nanoparticles had a size range of 10.1–18.8 nm (Figure 4B). The MnO_2_ nanoparticles were irregularly spherical, with a size range of 19.8–63.9 nm (Figure 4C). The SEM of the bulk metal oxides is shown in Figures 4D–F. Bulk ZnO had a size range of 393.6–415.0 nm (Figure 4D), while MgO had a mixed population that was irregularly shaped to circular with a size range of 160.7–284.6 nm (Figure 4E). On the other hand, the MnO_2_ bulk was aggregated which were closely compacted together with a size of 278.1 nm (Figure 4F).

### *In vitro* Antimicrobial Activity of Metal Oxide NPs

**FIGURE 5:**
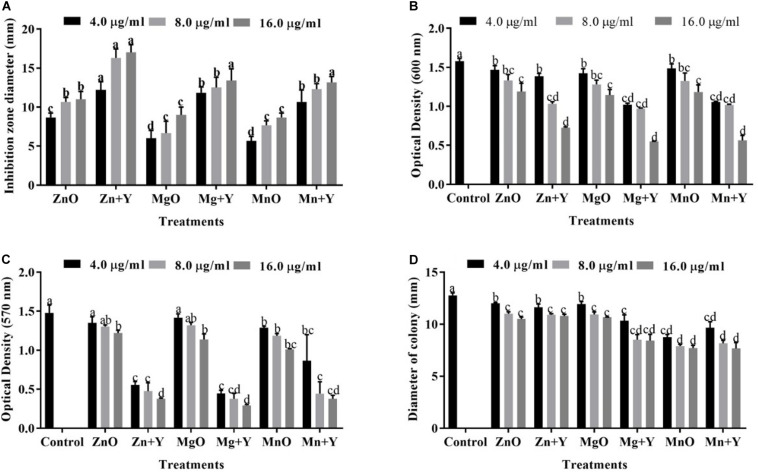
**(A)** Inhibitory effect of bulk metal oxide and nanoparticles. **(B)** Effect of the bulk metal oxide and nanoparticles on bacterial growth. **(C)** Effect of the bulk metal oxide and nanoparticles on biofilm formation. **(D)** Effect of the bulk metal oxide and nanoparticles on the swarming motility of *Xoo* strain GZ 0006. Zn + Y, ZnO nanoparticles; Mg + Y, MgO nanoparticles; Mn + Y, MnO_2_ nanoparticles; ZnO, Bulk ZnO; MgO, Bulk MgO, MnO, Bulk MnO_2_. Values are mean ± standard deviation of three replicates and bars with the same letters are not significantly different in LSD test (*P* < 0.05).

The antibacterial performance of ZnO, MnO_2_, and MgO nanoparticles synthesized by *P. polymyxa* strain Sx3 were tested against *Xoo* strain GZ 0006 (Figure 5A). ZnO nanoparticles (16.0 μg/ml) resulted in an average clearing diameter of 17.0 mm, which was significantly different from the treated concentration of 4.0 μg/ml. The MgO nanoparticles had an average clearing diameter of 13.4 mm, and MnO_2_ nanoparticles had an average clearing diameter of 13.2 mm, which was significantly different from the concentration of 4.0 μg/ml (Figure 5A). The bulk metal oxides at 16.0 μg/ml had an average clearing diameter of 11.0, 9.0, and 9.0 mm, respectively, for ZnO, MgO, and MnO_2_ which was significantly different from the synthesized nanoparticles (Figure 5A).

### Biofilm, Bacterial Growth Inhibition and Swarming Motility

The growth of *Xoo* strain GZ 0006 was negatively affected wand OD_600_ significantly reduced in the presence of ZnO, MnO_2_, and MgO nanoparticles (Figure 5B). The minimum inhibitory concentration (MIC) for each treatment was determined in a 96-well microtiter plate at different concentration (4.0, 8.0, and 16.0 μg/ml) (Table 1). For all the nanoparticles, the maximum bacterial growth inhibition (OD_600_) occurs at 16.0 μg/ml (Figure 5B), for which OD_600_ reduction of 54.0, 64.3, and 65.1% for ZnO, MnO_2_, and MgO nanoparticles, respectively. On the other hand, the bacterial interaction with the bulk metal oxides caused OD_600_ reduction of 24.5, 27.5, and 24.9%, respectively, for ZnO, MgO, and MnO_2_ (Figure 5B). In detail, the maximum OD_600_ for control without nanoparticle was 1.58, and the data reduced to 0.72, 0.56, and 0.55 with the addition of ZnO, MnO_2_, and MgO nanoparticles, respectively (Figure 5B), while the bulk metal oxides reduced the bacterial number to 1.19, 1.14, and 1.18, respectively, for ZnO, MgO, and MnO_2_ at 16.0 μg/ml (Figure 5B).

**TABLE 1:** The minimum inhibitory concentration (MIC) of ZnO, MgO, and MnO_2_ nanoparticles.

Nanoparticles	MIC (μg/ml) ± SD
ZnO nanoparticles	3.5 ± 0.5*
MgO nanoparticles	3.5 ± 0.9
MnO_2_ nanoparticles	3.6 ± 0.6

*Values are the mean ± SD of three replicates.

Biofilm formation (OD_570_) of *Xoo* strain GZ 0006 was significantly reduced with the application of nanoparticles (4.0, 8.0, and 16.0 μg/ml) (Figure 5C). The biofilm production was significantly reduced at 16.0 μg/ml by 74.5, 74.4, and 80.2% for ZnO, MnO_2_, and MgO nanoparticles, respectively (Figure 5C), while the bulk metal oxides reduced *Xoo* biofilm formation at 16.0 μg/ml by 17.5, 23.1, and 31.7%, respectively, for ZnO, MgO, and MnO_2_ (Figure 5C). *Xoo* strain GZ 0006 swam in the semi-solid agar medium and had an average diameter of 12.8 mm, 24 h post-incubation (Figure 5D). Treatment of the semi-solid agar medium with ZnO, MnO_2_, and MgO nanoparticles significantly reduced the swarming motility of *Xoo* at all tested concentrations. The swarming motility of *Xoo* was decreased by 33.9, 40.0, and 39.7%, respectively, for ZnO, MnO_2_, and MgO nanoparticles treatment (Figure 5D). The bulk metal oxides reduced the swarming motility of *Xoo* at 16.0 μg/ml by 17.8, 15.5, and 16.4%, respectively, for ZnO, MgO, and MnO_2_ (Figure 5D).

### Damage of Bacterial Cells by the Metal Oxide NPs

**FIGURE 6:**
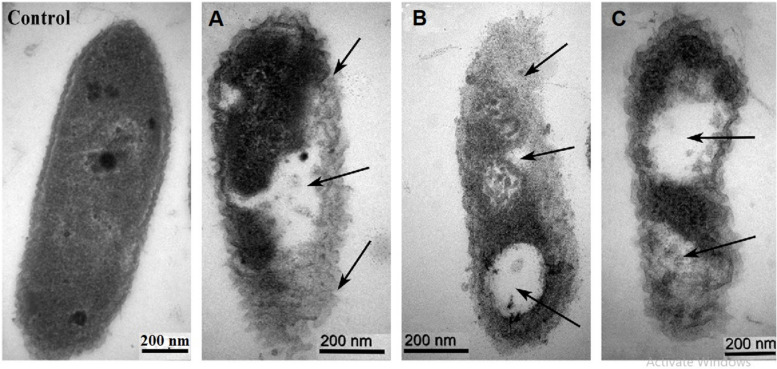
TEM images of *Xoo* strain GZ 0006 cells treated with **(Control)** double distilled water **(A)** ZnO nanoparticles **(B)** MgO nanoparticles **(C)** MnO_2_ nanoparticles. Magnification 100,000X for Control; 120,000X for **(A,B)**; 150,000X for **(C)**. The arrows indicate the morphological damages of the cell and empty cell content.

Transmission electron micrographs of *Xoo* strain GZ 0006 treated with ZnO, MnO_2_, and MgO nanoparticles are shown in Figure 6. The *Xoo* strain GZ 0006 treated with double distilled water (control) in Figure 6 shows the well-defined cell membrane and the evenly stained interior of the cell, which corresponds to the proteins and DNA of the cell (Stoimenov et al., 2002). Treatment of the cell with ZnO, MnO_2_, and MgO nanoparticles caused cellular changes in the morphology, from which the nanoparticles had successfully penetrated the cell, thus damaging the cell membrane and caused cellular content leaking out (Figures 6A–C).

### ROS Production

**FIGURE 7:**
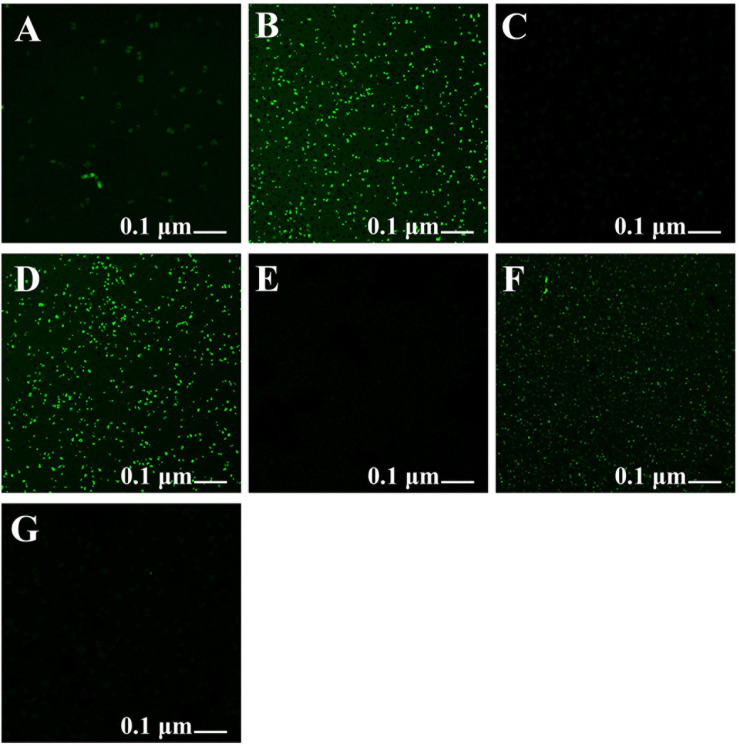
Formation of reactive oxygen species in Xoo strain GZ 0006 cells after 4 h incubation period with **(A)** Double distilled water, **(B)** ZnO nanoparticles, **(C)** Rotenone before ZnO nanoparticles, **(D)** MgO nanoparticles, **(E)** Rotenone before MgO nanoparticles, **(F)** MnO_2_ nanoparticles, **(G)** Rotenone before MnO_2_ nanoparticles treatment.

The *Xoo* strain GZ 0006 treated with rotenone along with the respective nanoparticles inhibited the production of reactive oxygen species (ROS), which was similar to the control (Figures 7A,C,E,G), while treatment with ZnO, MnO_2_, and MgO nanoparticles caused a high degree of fluorescent intensity (Figures 7B,D,F). The high fluorescent intensity observed in the bacteria treated with the nanoparticles after incubation in the dark confirms the death of the bacteria as a consequent for the ROS production (Figures 7B,D,F).

### Effect of Nanoparticles Treatment on Plants Infected by *Xoo* Strain GZ 0006

Inoculation of rice leaves by leaf clipping with *Xoo* strain GZ 0006 caused percentage diseased leaf area (%DLA) of 74.7% (Table 2). The%DLA was significantly reduced to 18.0, 27.5, and 20.1%, respectively, when the rice seedlings were sprayed with ZnO, MgO, and MnO_2_ nanoparticles (Table 2). A significant decrease of 41.8, 40.2, 53.85, and 57.89%, respectively, in shoot length, root length, fresh and dry weight was caused as a result of inoculating the rice seedlings with *Xoo* as compared to the plants treated with double distilled water (control) (Table 2). Foliar treatment of plants infected with *Xoo* with 16.0 μg/ml ZnO, MgO, and MnO_2_ nanoparticles significantly increased the growth and biomass of the rice plants when compared to the infected pathogen plants (Table 2). Plants treated with 16.0 μg/ml ZnO, MgO, and MnO_2_ nanoparticles alone had a significant increase in shoot length, root length, fresh weight, and dry weight compared to the control treated with double distilled water (Table 2). However, treatment with ZnO nanoparticles alone had the highest significant increase of 26.8, 38.7, 61.0, and 60.4% in shoot length, root length, fresh and dry weight, respectively, when compared to plants treated with double distilled water (Table 2).

**TABLE 2:** *In vivo* inhibitory effect of ZnO, MgO, and MnO_2_ nanoparticles on *Xoo* strain GZ 0006.

Treatments	Shoot length (cm)	Root length (cm)	Fresh weight (g)	Dry weight (g)	% DLA
ddH_2_O	33.50 ± 3.20^c^*	11.70 ± 0.62^*d*^	1.56 ± 0.06^*d*^	0.19 ± 0.07^*cd*^	–
ZnY	45.77 ± 1.65^*a*^	19.07 ± 0.24^*a*^	4.00 ± 0.17^*a*^	0.48 ± 0.04^*a*^	–
ZnY + Xoo	40.37 ± 0.86^*b*^	16.77 ± 0.32^*b*^	1.65 ± 0.13^*d*^	0.24 ± 0.02^*bc*^	18.03 ± 3.49^*b*^
MgY	32.27 ± 0.59^*cd*^	19.80 ± 0.36^*a*^	2.41 ± 0.20^*c*^	0.28 ± 0.04^*b*^	–
MgY + Xoo	29.37 ± 1.12^*d*^	11.33 ± 0.40^*de*^	1.55 ± 0.02^*d*^	0.21 ± 0.02^*bcd*^	27.46 ± 6.02^*b*^
MnY	39.70 ± 1.55^*b*^	14.20 ± 0.72^*c*^	3.31 ± 0.27^*b*^	0.43 ± 0.05^*a*^	–
MnY + Xoo	24.50 ± 3.39^*e*^	10.53 ± 0.70^*e*^	1.55 ± 0.13^*d*^	0.14 ± 0.04^*de*^	20.10 ± 2.19^*b*^
Xoo	19.5 ± 0.90^*f*^	7.00 ± 0.20^*f*^	0.72 ± 0.09^*e*^	0.08 ± 0.02^*e*^	74.71 ± 6.65^*a*^

*Values are the mean ± SD of three replicates. Values with different letters are significantly different in LSD tests (P < 0.05). ddH_2_O, double distilled water; ZnY, ZnO nanoparticles; MgY, MgO nanoparticles; MnY, MnO_2_ nanoparticles; ZnY + Xoo, Xoo strain GZ 0006 infected plant with ZnO nanoparticles; MgY + Xoo, Xoo strain GZ 0006 infected plant with MgO nanoparticles; MnY + Xoo, Xoo strain GZ 0006 infected plant with MnO_2_ nanoparticles.

## Discussion

In this study, the synthesis procedure started from respective metal oxide rather than the salt precursor because of its relative non-toxicity as compared to salt precursor which are very toxic (Stoimenov et al., 2002; Jones et al., 2008; Sundrarajan et al., 2012). Metal oxides can also adopt a vast number of structural geometries with unique physical and chemical properties (Montero et al., 2010; Lakshmi et al., 2012; Mirzaei and Davoodnia, 2012; Divyapriya et al., 2014; Tang and Lv, 2014; Moon et al., 2015; Ogunyemi et al., 2019a). The synthesis protocol used in this study was adopted because it is cheap and eco-friendly. In addition, the protocol used enhanced the uniformity of shapes of the synthesized nanoparticles as opposed the findings of Abdallah et al. (2020), who reported diverse polymorphic shapes. Ogunyemi et al. (2019b) assert that the small size of ZnO nanoparticles synthesized by Olive leaves played an important role in the strong antibacterial activity recorded against rice pathogen. Therefore, in biosynthesis study it is essential to adopt a protocol that produces small-sized nanoparticles. The protocol adopted in this study produced small-sized nanoparticles < 63 nm as compared to the size range of 200–350 nm reported by Ilk et al. (2016). The mechanism of synthesis of the nanoparticles by *P. polymyxa* which served as biosurfactant involves the absorption onto Zn, Mg, and Mn forming complexation. This is followed by nucleation, which is as a result of the binding of Zn, Mg, and Mn ions by surfactin carboxyl group present in the culture filtrate of *P. polymyxa* Sx3. The aggregation of the surfactant molecules in solution resulted in localization of negative functional groups which enriched the Zn, Mg, and Mn ions via inter- and intra-molecular bridges (Bastrzyk et al., 2019). This is followed by formation of crystals via Ostwald ripening, the formed crystal proceed with crystallization via the coalescence mechanism forming layered, porous structures characterized by higher specific surface areas (De Yoreo et al., 2015; Polowezyk et al., 2016).

The UV-Vis spectroscopy is mostly used for characterizing the optical properties and electronic structure of nanoparticles; the absorption peaks are concerning the size of the nanoparticles (Philip, 2008). It is an important technique used to confirm the presence of NPs in an aqueous solution (Philip, 2008). The absence of peaks in the bulk solutions of ZnO, MgO, and MnO_2_ indicates the absence of nanoparticles in the bulk solutions which is consistent with the reports of Abdallah et al. (2020). The UV peak of 385, 230, and 230 nm, respectively, for ZnO, MgO, and MnO_2_ nanoparticles recorded in this study is consistent with our previous study of biosynthesis of MgO and MnO_2_ nanoparticles (Ogunyemi et al., 2019a), while Abdallah et al. (2019b, 2020) recorded a different peak for ZnO and MgO nanoparticles. In the study of Salunke et al. (2015), a peak of 365 nm was observed for MnO_2_ nanoparticles. The difference in the peaks reported maybe as a result of the different biosynthesis protocols used.

The functional groups observed in the synthesized nanoparticles confirmed that the culture filtrate of *P. polymyxa* strain Sx3 acted as capping agent while the protein functional group contained in the culture filtrate reduced the metal ions for the synthesis of ZnO, MgO, and MnO_2_ nanoparticles. The sharp peak of 3,699 cm^–1^ observed is probably as a result of the single coordinated hydroxides contained in the nanoparticles, which acts as proton acceptor on the surface of the nanostructured MgO powders (Kwon and Park, 2009). The functional group C–O, C–O–H, C–N, N–H, and C = C contained in all the nanoparticles is the amino acid residues and protein synthesized. Proteins play a vital role in the mode of application, which is responsible for the biosynthesis of nanoparticles (Lakshmi et al., 2012; Padman et al., 2014; Fouad et al., 2017). Therefore, with the functional groups observed in all the nanoparticles, we conclude that the protein in the cell-free supernatant played an active role in the reduction of the nanoparticles. Involvements of other biomolecules such as enzymes, esters, anhydrides, and alkynes in the cell wall of bacteria have been reported to synthesize nanoparticles (Janeiro et al., 2005; Udayasoorian et al., 2011). The formation of ZnO, MgO, and MnO_2_ NPs occur due to the interactive involvement of protein and other functional groups of *P. polymyxa* secondary metabolites and enzymes in its reduction. This agrees with the previous report that the protein component of the secondary metabolite of pyoverdine in *P. aeruginosa*, enzyme nitrate reductase (Kalimuthu et al., 2008), or protein in the cell-free supernatant of *Bacillus* sp. was responsible for silver nanoparticles formation (Rangarajan et al., 2018).

The SEM micrographs represent visual evidence of the change in size and morphology between the bulk and the nanoparticles. It is inferred that *P. polymyxa* treatment on the metal oxides (ZnO, MgO, and MnO_2_) may serve as biosurfactant, which acted as stabilizing and reducing agent while preventing the formation of aggregates (Kiran et al., 2010). The *P. polymyxa* also, may act as enhancers in the synthesis of the nanoparticles. Metabolite lipopeptides from *P. polymyxa*, which are natural surfactants, acted as a stabilizing agent in silver nanoparticles synthesis and reduced the formation of aggregates due to the electrostatic force of attraction thereby, facilitating uniform morphology of nanoparticles (Kiran et al., 2010; Rangarajan et al., 2018). Diverse shapes of mixed population containing hexagonal, spherical, and disc-shaped have been reported for ZnO, MgO, and MnO_2_ nanoparticles (Omid et al., 2014; Sharma P. et al., 2017; Abdallah et al., 2020), while in this study a uniformity in the shapes of the synthesized nanoparticles were reported.

The sizes of the samples by SEM were in the range of 56.1–110.0 nm for ZnONPs, 10.1–18.8 nm for MgONPs, and 19.8–63.9 nm for MnO_2_NPs. The average dominant crystallite sizes by the XRD calculated from the diffraction peaks by Scherrer’s equation were 62.8, 18.8, and 10.9 nm for ZnONPs, MnO_2_NPs, and MgONPs, respectively, thus, the SEM results are similar with that of XRD. Similar average crystallite size results were reported by Abdallah et al. (2020), who synthesized ZnONPs by using green tomato fruits. Also, the results in this study are in agreement with previous study of Ogunyemi et al. (2019a), who reported similar average size of 16.5 and 18.2 nm, respectively, for MnO_2_NPs and MgONPs when synthesized by Chamomile flower extract, whereas Salunke et al. (2015) reported an higher average size of 34.4 nm for MnO_2_NPs. The SEM-EDS shows the elemental analysis of the nanoparticles contained only the element and oxygen which confirms the presence of the nanoparticles in the samples and its high purity. The elemental percentage of ZnONPs in this study is consistent with the reports of Abdallah et al. (2020), while those of MnO_2_NPs and MgONPs are in agreement with the report of Ogunyemi et al. (2019a). Upon the treatment with ZnO, MgO, or MnO_2_, the characteristic cell damage and leakage of cell content observed using TEM agrees with the reports of Stoimenov et al. (2002), who reported the loss of distinctive cell membrane of *Escherichia coli.*

After 18 h of incubation, growth inhibition zones were observed from the spot of treatment with the nanoparticles, the appearance of growth inhibition zones signified the inability of the *Xoo* to grow around the area of the respective nanoparticles application. The nanoparticles exhibited substantial antibacterial performance against *Xoo* strain GZ 0006. The higher significant growth inhibition zones observed in the treatment with the synthesized nanoparticles at all concentrations as against the bulk metal oxide in this study is in consistence with the recent report of Abdallah et al. (2020). Interaction of metallic nanoparticles with bacteria caused bacterial surface damage and has been proposed as an explanation for the antibacterial activity (Stoimenov et al., 2002; Makhluf et al., 2005). This interaction explains the reduction in biofilm formation and swarming motility observed in this study. The inhibition of biofilm formation by the synthesized nanoparticles at 8.0 and 16.0 μg/ml were not significantly different from each other while the bacterial number and swarming motility (16.0 μg/ml) of MgO and MnO_2_ nanoparticles were significantly difference from ZnONPs. However, ZnONPs at 8.0 and 16.0 μg/ml had the highest growth inhibition zone which was significantly different from MgO and MnO_2_ nanoparticles.

It is well known that bacterial numbers significantly affected the quantity of biofilm, but may not motility, thus, it could be speculated that the reduction in biofilm formation may be mainly due to the reduced numbers of bacterial cells (Abdallah et al., 2019a). In contrast, out results revealed a greater reduction in biofilm formation than that of bacterial number, while the motility was also marked reduced by biogenic NPs. Therefore, it could be inferred that the antibacterial activity of biogenic NPs may be, at least partially, due to the reduced biofilm formation and motility. Interestingly, the bacterial virulence has been reported to be highly dependent on the biofilm formation and swarming motility (Ogunyemi et al., 2019d), hence, the significant reduction in biofilm formation and swarming motility contribute to the effective suppression of BLB disease by nanoparticle.

The growth of *Xoo* was reduced with increasing concentrations of the nanoparticles, which is in agreement with the studies of nanoparticle concentration-dependent antibacterial activity (Jin and He, 2011; Sharma G. et al., 2017; Duffy et al., 2018). In contrast, the antibacterial activity of the bulk metal oxides (ZnO, MgO, and MnO_2_) was of minimal effect compared to their nanoparticles counterparts. Previous researchers have reported nanoparticles to have strong antibacterial activity against *Xoo* which is consistent with our study (Li et al., 2016; Ibrahim et al., 2019). The nanoparticles with high surface to volume ratio enhanced the number of reaction sites, which increased the antibacterial activity compared to the bulk metal oxides (Stoimenov et al., 2002; Makhluf et al., 2005). Also, the nano-dimension (1–100 nm) of ZnO, MgO, and MnO_2_ nanoparticles benefits the bacterial membrane penetration, which enhanced their antibacterial activity compared to the bulk counterparts whose sizes were greater than 100 nm (Ogunyemi et al., 2019b).

The substantial antibacterial performance of the nanoparticles observed in this study is related to ROS production. It can, therefore, be proposed that the *Xoo* cell internalizes the nanoparticle ions, which invariably inhibited respiratory enzymes, arrested the bacterial growth, and facilitated the production of reactive oxygen species, which lead to cell damage and leakage of cytoplasmic materials. This result is consistent with Cai et al. (2018), who reported ROS production when *Ralstonia solanacearum* cell was treated with MgO nanoparticles. ROS production increased the oxidative stress in cells, which invariably cause DNA, protein, and cell damage (Le Ouay and Stellacci, 2015; Abdallah et al., 2020). The antibacterial activity of nanoparticles in the dark as observed in this study due to ROS production, was also reported by Hirota et al. (2010) when ZnO nanoparticles were tested against *Escherichia coli*. Also, Cai et al. (2018) reported the generation of ROS at a wavelength of 488 nm after 30 min of incubation in the dark. Adams et al. (2006) found that the inhibitory effect of nanoparticles on bacteria due to ROS production could occur both under light and dark conditions producing superoxide ions, which is consistent with the findings of Jones et al. (2008). Therefore, with such consistency in ROS production a further mechanism is involved in its production in the dark.

Recently, nanoparticles treatment have been reported as an efficient alternative in disease-resistant pathogens control measure (Cai et al., 2018), however, its use has been limited due to the toxic effect which has been reported as the major concern when using nanoparticles as a control measure (Wang et al., 2016). Therefore, in order to address this concern and optimize its use as efficient control measure, low concentrations of the synthesized ZnO, MgO, and MnO_2_ nanoparticles were used. Nanoparticles at low concentrations are safe and can effectively increase agronomic and crop productivity (Tarafdar et al., 2014; Zabrieski et al., 2015; Liu et al., 2016). The positive significant increment in shoot length, root length, fresh and dry weight recorded in this study as a result of ZnO, MgO, and MnO_2_ nanoparticles application is in conformity with previous researchers, who reported that different nanoscale metal oxide particles enhanced plant growth and improved crop yield (Tarafdar et al., 2014; Raliya et al., 2015; Rathore and Tarafdar, 2015; Cai et al., 2018).

Treatment of *Xoo* infected plants with ZnO, MgO, and MnO_2_ nanoparticles decreased the BLB disease leaf area *in vivo.* The ability of the synthesized nanoparticles to suppress bacterial leaf blight expression is as a result of its close interaction with the plant pathogen. The significant bacterial disease reduction by the nanoparticles treatment in this study is in conformation with Cai et al. (2018) study of bacterial wilt diseases suppression on tobacco plants when treated with MgO nanoparticles. Metallic nanoparticles of ZnO, MgO, and MnO_2_ at safe dosage have been reported to be non-toxic, bio-safe, and bio-compatible (Hameed et al., 2016; Cai et al., 2018). Therefore, the application of ZnO, MgO, and MnO_2_ nanoparticles on rice plants in this study serves to increase the growth parameters and biomass of rice while reducing the diseases expression of rice bacterial leaf blight.

## Conclusion

In the current study, we have successfully synthesized ZnO, MgO, and MnO_2_ nanoparticles using rhizophytic bacteria *P. polymyxa* strain Sx3. It is an eco-friendly, bio-compatible and cost-effective method. To the best of the author’s knowledge, this is the first report of using rhizophytic bacteria, *P. polymyxa*, to green synthesize ZnO, MgO, and MnO_2_. The biogenic nanoparticles showed strong antibacterial, anti-biofilm, and anti-swarming activities as compared to the bulk metal oxide counterparts against *Xoo* strain GZ 0006. In addition, the nanoparticle application effectively suppressed BLB disease expression and positively increased the growth and biomass of rice seedlings when tested *in vivo.* Therefore, it confirms its potential as an active antimicrobial agent against this infectious pathogen of BLB. Overall, the nanoparticles synthesized by *P. polymyxa* could be adopted as an alternative antimicrobial agent for managing disease-resistant pathogens in the future.

## Data Availability Statement

The raw data supporting the conclusions of this article will be made available by the authors, without undue reservation.

## Author Contributions

SO, MZ, TA, and BL: conceptualization. BL: methodology, supervision, and funding acquisition. SO: software, formal analysis, and writing—original draft preparation. WQ, YA, and TA: validation. MA: investigation. CY: resources. MZ: data curation. WQ and TA: writing—review and editing. YY: visualization. JC: project administration. All authors contributed to the article and approved the submitted version.

## Conflict of Interest

The authors declare that the research was conducted in the absence of any commercial or financial relationships that could be construed as a potential conflict of interest.
